# Anaerobes in clean orthopedic surgery? Is it a problem?

**DOI:** 10.1186/2047-2994-4-S1-P71

**Published:** 2015-06-16

**Authors:** I Uckay, B Kressmann, A Agostinho, C Landelle, M Al-Mayahi, D Pittet

**Affiliations:** 1 Geneva University Hospitals, Geneva, Switzerland; 2 Infection Control Program, Geneva University Hospitals, Geneva, Switzerland

## Introduction

Scheduled orthopedic interventions are considered as “clean surgery”, unless the intervention follows open fracture injury-related conditions or in the presence of spontaneous soft tissue infection.

## Objectives

To investigate whether some patient populations and types of surgery would be at particular risk for anaerobic infections.

## Methods

Retrospective cohort study of adult in-patients operated for orthopedic infections from 2004-2014. We assessed obligate anaerobes and considered only first clinical infection episodes; thus possible recurrent infections were excluded.

## Results

Anaerobes, isolated from intraoperative samples, were identified in 65 (2.4%) of a total of 2740 surgical procedures. Anaerobes were identified in half (33/65; 51%) as responsible for monomicrobial infections. Anaerobic co-infections were particularly related to plates, mostly in the lower extremities and to open fractures (8/150 vs. 57/2590; *p*=0.01) and polymicrobial infections (33/572 vs. 32/1853; *p*<0.01). In contrast, anaerobes were never documented in septic bursitis, and less likely in native bone or prosthetic joint infections (7/321; 2%). Anaerobic infection was also less frequent in immune-suppressed patients, including diabetic patients, with overall incidence of infection of 1.0% and 0.9%, respectively. The serum C-reactive protein level at admission was lower for infections involving anaerobes (median 61 mg/L vs. 77 mg/L, *p*=0.04) than for infections that did not involve anaerobes. By multivariate analysis adjusting for the case-mix, the presence of fracture-devices such as plates (odds ratio 2.1, 95% CI 1.3-3.5) was the only variable positively associated with anaerobic infection, while underlying immune suppression yielded to be formally protective (OR 0.4, 0.2-0.8). Sex, age, the presence of abscess formation, type of material, and exposure to antibiotic therapy prior to intraoperative sampling showed no association.

## Conclusion

Obligate anaerobes in orthopedic surgery are co-pathogens in half of the cases and mostly encountered in infections of the lower extremity. However, most of these infections are not classical surgical site infections, but often related to open fractures or severe trauma.

## Disclosure of interest

None declared.

